# Emerging role of bacterial outer membrane vesicle in gastrointestinal tract

**DOI:** 10.1186/s13099-023-00543-2

**Published:** 2023-04-27

**Authors:** Cheng-mei Tian, Mei-feng Yang, Hao-ming Xu, Min-zheng Zhu, Yuan Zhang, Jun Yao, Li-sheng Wang, Yu-jie Liang, De-feng Li

**Affiliations:** 1 Department of Emergency, Shenzhen People’s Hospital (The Second Clinical Medical College, Jinan University; the First Affiliated Hospital, Southern University of Science and Technology), Shenzhen, 518020 Guangdong China; 2 Department of Hematology, Yantian District People’s Hospital, Shenzhen, Guangdong China; 3 Department of Gastroenterology and Hepatology, Guangzhou Digestive Disease Center, Guangzhou First People’s Hospital, School of Medicine, South China University of Technology, Guangzhou, China; 4 Department of Medical Administration, Huizhou Institute of Occupational Diseases Control and Prevention, Huizhou, Guangdong China; 5 Department of Gastroenterology, Shenzhen People’s Hospital (The Second Clinical Medical College, Jinan University; the First Affiliated Hospital, Southern University of Science and Technology), No.1017, Dongmen North Road, Luohu District, Shenzhen, 518020 People’s Republic of China; 6 Department of Child and Adolescent Psychiatry, Shenzhen Kangning Hospital, No.1080, Cuizu Road, Luohu District, Shenzhen, 518020 People’s Republic of China

**Keywords:** Bacterial, Microbiota, Outer membrane vesicle, Extracellular vesicles, Gastrointestinal tract

## Abstract

Bacteria form a highly complex ecosystem in the gastrointestinal (GI) tract. In recent years, mounting evidence has shown that bacteria can release nanoscale phospholipid bilayer particles that encapsulate nucleic acids, proteins, lipids, and other molecules. Extracellular vesicles (EVs) are secreted by microorganisms and can transport a variety of important factors, such as virulence factors, antibiotics, HGT, and defensive factors produced by host eukaryotic cells. In addition, these EVs are vital in facilitating communication between microbiota and the host. Therefore, bacterial EVs play a crucial role in maintaining the GI tract’s health and proper functioning. In this review, we outlined the structure and composition of bacterial EVs. Additionally, we highlighted the critical role that bacterial EVs play in immune regulation and in maintaining the balance of the gut microbiota. To further elucidate progress in the field of intestinal research and to provide a reference for future EV studies, we also discussed the clinical and pharmacological potential of bacterial EVs, as well as the necessary efforts required to understand the mechanisms of interaction between bacterial EVs and gut pathogenesis.

## Introduction

The gastrointestinal (GI) tract serves as the primary site for food digestion and absorption while also acting as a gateway for toxin invasion. It performs the dual functions of digestion and absorption as well as intestinal defense, which cannot be accomplished by the digestive system alone [1]. Hidden within the GI tract is a unique "organ" called the intestinal flora, which plays a critical role in digesting chyme and protecting the intestines [2–4]. The intestinal flora consists of thousands of species and over 10 trillion bacteria, which lack a cell connection and stable extracellular matrix with host cells. As a result, communication between surface proteins and secretory proteins becomes challenging. Extracellular vesicles (EVs) produced by intestinal bacteria serve as the primary mode of material transport and communication [5], and they remain active in the intestinal microenvironment while passing through the vascular barrier to enter the bloodstream [6]. Using specific surface structures, EVs recognize and bind to either bacteria or host cells, transmitting substances and signals. Intestinal bacteria and host cells continuously secrete and accept extracellular vesicles in the intestinal microenvironment, thereby affecting the viability of bacteria and the physiological function of host cells. These vesicles transport substances and information between the two types of cells [5]. In recent years, mounting evidence has confirmed that bacteria can secrete EVs as phages, antibiotics, and eukaryotic host defense actors [7, 8]. Additionally, EVs play crucial roles in intercellular communication, virulence factor transport, horizontal gene transfer (HGT), nutrient and electron transport, and biofilm formation [9–11]. Detecting bacterial EVs enables us to understand the composition of intestinal flora and aid in diagnosis. Transforming bacterial EVs can help us develop bacterial vaccines or immune adjuvants, as well as target drug delivery. However, future work should focus on regulating intestinal flora and protecting host cells through bacterial EV preparations [12, 13].

## Composition and distribution of the microflora in the gut

**Fig. 1:**
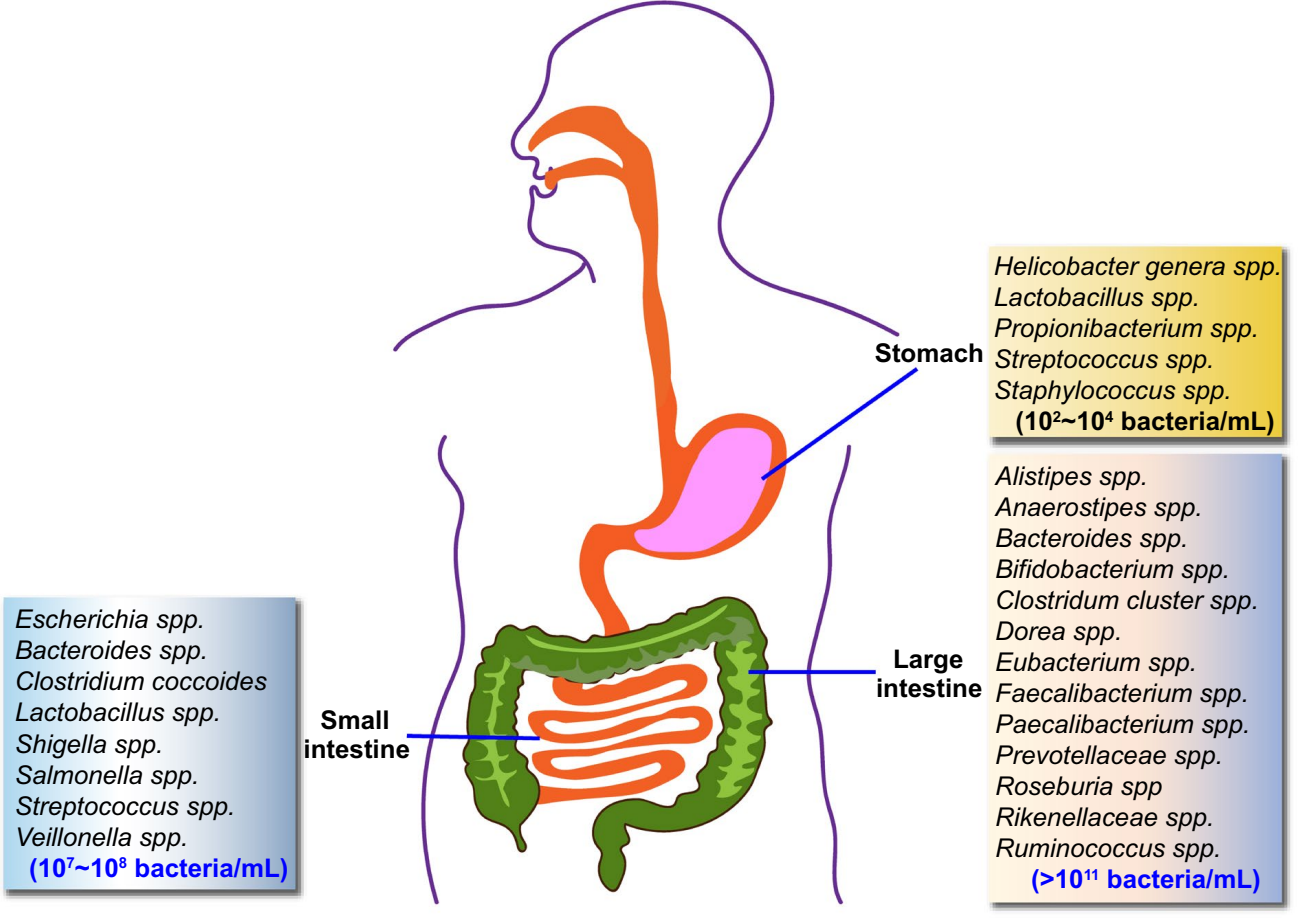
The bacterial flora inhabits in regions of human gastrointestinal tract constitute a complex ecosystem. More than 10 [14] microorganisms, 500 bacteria species have been identified in GI. The upper gastrointestinal tract (stomach, duodenum, jejunum, and upper ileum) is usually contained *Lactobacillus*, with bacterial concentrations less than 10 [4] microorganisms/ml. By contrast, bacteria in the large intestine are dramatic increase as 10 million bacteria. *Anaerobic bacteria (A. bacteria)* such as *Bacteroides*, *Enterobacter*, *Anaerobic Streptococcus (A. Streptococcus)*, *Clostridium* and *Lactobacillus* are 1000 times more abundant than facultative anaerobes such as *E. coli*. It is a general trend that bacteria increase in complexity and concentration as they enter the gastrointestinal tract

More than 99% of the microbiota in the intestine is composed of intestinal flora, and the number of bacteria in the intestine exceeds the number of human cells. There are at least 1000 to 150 species present in the intestinal tract, and more than 500 species can be cultured. These are concentrated in 5–7 bacterial phyla, of which Bacteroidetes and Firmicutes make up about 95% and are part of the predominant microflora (Fig. 1) [14]. Typically, these bacteria are obligate anaerobes and specialize in colonizing the intestine. Most of them are probiotics and have a symbiotic relationship with the host [15]. However, most of the sub-dominant microflora belong to foreign or transient flora with high mobility. They are mainly aerobic bacteria or facultative anaerobic bacteria and may have potential pathogenicity that can cause harm to the host [15]. The intestinal flora is complex and diverse, and its composition can be influenced by various factors, such as age, diet, exercise, heredity, drugs, living environment, regional climate, and health status [3, 4, 15–20]. The leading indicators for evaluating intestinal flora include the concentration of colonic bacteria, the diversity of intestinal bacteria, and the ratio of probiotics [21]. In healthy individuals, probiotics are usually the dominant bacterial species in the intestine. Studies have shown that the proportion of probiotics in the intestine is about 70% in healthy individuals, 25% in average individuals, and 15% in those with constipation. However, the proportion of probiotics is only about 10% in the intestine of cancer patients [21, 22]. Figure 1 provides a visual representation of the microbial density and species present in the gut.

The intestinal microbiota is closely intertwined with the physical and chemical environment of the gut and the host cells, collectively constituting the intestinal micro-ecosystem. These elements have a reciprocal and restrictive influence on each other, always striving to maintain a dynamic equilibrium, known as intestinal microecological homeostasis [23]. An imbalance in the intestinal flora can disrupt the host cells and the physical and chemical environment of the intestine, leading to intestinal micro-ecosystem disorders. This imbalance can cause not only acute and chronic inflammatory reactions, GI dysfunction, digestive tract tumors, and other digestive system diseases but also extraintestinal diseases, such as obesity, type 2 diabetes, liver disease, atherosclerosis, infectious diseases, allergic diseases, and mental and neurological dysfunction [24].

## Biogenesis of bacterial EVs

Vesicle transport is the primary means by which cells transport macromolecules. EVs are a diverse array of vesicles released by cells [25–28]. Bacteria are unicellular prokaryotes. Bacterial EVs are secreted and transported by bacteria, facilitating the transfer of information and energy conversion. This mode of transport differs from small molecule transmembrane transport and the bacterial protein I–IX secretion systems [27, 29–31].

**Fig. 2:**
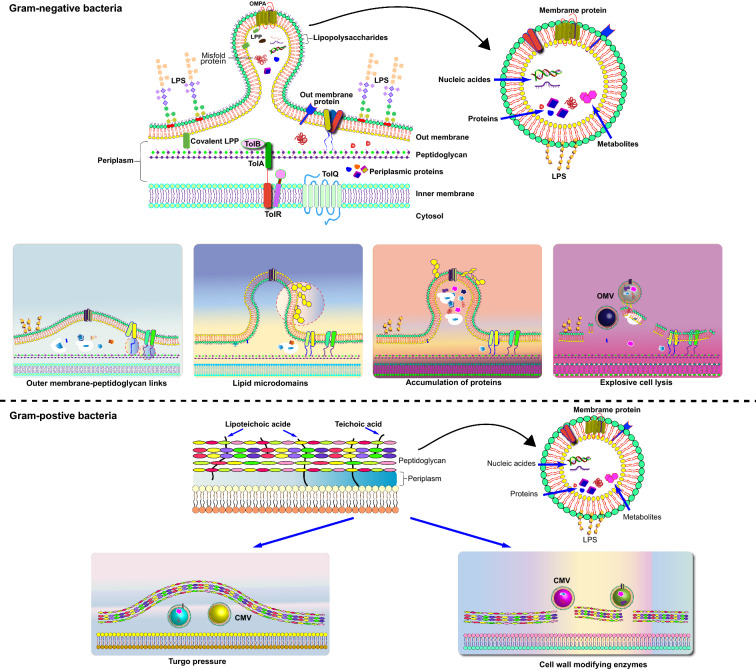
Biogenesis model and composition of bacterial extracellular vesicles (bEVs). The composition of bEVs includes biphospholipid layers, proteins, glycoproteins, metabolites, and nucleic acids. **A** bEVs derived from Gram-negative bacteria can be released through the outer membrane; (i) by reducing outer membrane-peptidoglycan protein linkages; (ii) lipid/LPS differential assembly in specific regions of the outer membrane; (iii) swelling pressure by accumulation of protein or peptidoglycan fragments in the periplasmic space; (iiii) blast by cell lysis. **B** bEVs derived from Gram-positive bacteria can be released from the swelling pressure caused by EV accumulation through the cell wall composed of peptidoglycan; these turgor pressure promotes membrane curvature, then bEVs are released by peptidoglycan-degrading enzymes

Bacteria can be categorized into Gram-negative and Gram-positive based on Gram staining, and they differ in their secretion of EVs [27, 32, 33]. Gram-negative bacteria possess an outer membrane structure, and it is commonly believed that EVs are vesicles formed by the extrusion of the bacterial outer membrane, known as outer membrane vesicles (OMVs) [34, 35]. Most Gram-negative bacteria secrete OMVs, and it is believed that the size of bacterial OMVs is similar to that of eukaryotic microvesicles, resulting from the pinching off of the outer membrane [36]. The specific mechanism of exfoliation is not clear, but proposed models mainly include the dissociation of stable cross-links of cell walls, the enlargement of the distance between the inner and outer membranes of bacteria, the local bulging and breakage of the outer membranes, and the orderly exfoliation of OMVs under the regulation of bacterial genes [32, 33, 37–39]. Moreover, a small proportion of cells are lysed to form outer-inner membrane vesicles (OIMVs) and explosive outer membrane vesicles (EOMVs) [28, 40]. Cell lysis can be triggered by DNA damage or by the partial degradation of the peptidoglycan layer of the cell wall by autolysin to form pores, where the inner and outer membranes protrude outward to wrap the cytoplasmic components, forming vesicles that are eventually squeezed out of the bacterial surface to form OIMVs [40]. When cell death and lysis occur, membrane fragments produced by explosive lysis can re-aggregate and randomly encapsulate cytoplasmic components to form vesicles, known as EOMVs [41]. Figure 2 provides further details on the occurrence of OMVs, OIMVs, and EOMVs.

On the other hand, the cell wall of Gram-positive bacteria lacks an outer membrane structure and is encased with a thick peptidoglycan layer. Currently, it is widely accepted that the weakening of the peptidoglycan layer by cell wall degrading enzymes and the increase of bacterial internal pressure allow for the release of the bacterial inner membrane, and the bacterial plasma membrane wraps the cytoplasmic components and bulges outward to form vesicles known as cytoplasmic membrane vesicles (CMVs). Only a small fraction of Gram-positive bacteria secrete CMVs [27, 32, 42–47]. OMVs and CMVs differ in generation, morphology, and function, as summarized in Table 1 [48, 49]. OMVs and CMVs are collectively referred to as membrane vesicles (MVs) [29, 30]. While MVs and EVs secreted by eukaryotic cells share similarities in structure, they differ in composition and function, as outlined in Table 2 [43, 50].

**Table 1:** Comparison between gram-negative and gram-positive extracellular vesicles

Features	Gram-negative EVs	Gram-positive EVs	References
Origin	Outer membrane	Cytoplasmic membrane	[39]
Size	10 nm-300 nm	20 nm-400 nm	[39]
Components	Outer membrane proteins, periplasmic proteins, virulence factors, cytoplasmic proteins, inner membrane proteins, lipopolysaccharides, phospholipids, and peptidoglycan (10%-20%)	Cytoplasmic proteins, membrane-associated proteins, lipoteichoic acid (LTA), peptidoglycan (> 50%)	[39]
Genetic components	sRNA, mRNA, miRNA, luminal and surface associated DNA	sRNA, extracellular and chromosomal DNA	[39, 201]
Proteins	Outer membrane: OmpA, OmpC, OmpF, lipoprotein (Lpp), periplasmic: Alkaline phosphatase and AcrA	Single lipid membrane proteins: penicillin-binding, immunoglobulin G-binding (protein A), staphopain A, α-haemolysins, heat-shock protein	[42, 202]
Lipids	Glycerophopholipids, phosphatidylethanolamine, phophotidylglycerol and cardiolipin	Phosphatidylglycerol, myristic and palmitic acids	[120, 202]
Coagulation	E-selectin, P-selectin, thrombomodulin	Fibronectin binding protein, *staphylocoagulase precursor*, Vonwillebrand factor binding protein	[202]
Antibiotic resistance	β-lactamase, enzyme L5, multidrug efflux protein (*Mtr, Mex, TolC*)	β-lactamase, Penicillin-binding proteins: PBP1, PBP2, PBP2a, PBP3 and PBP4	[202]
Virulence factor delivery	Enzymes: phospholipase C, esterase lipase, alkaline phosphatase, serine protease Toxins: adenylatecyclase, cholera, cytolethal distending, PagJ, PagK1, VacA	InIB, LLO, IgG binding protein SbI, protective antigen, lethal factor, edema toxin, anthrolysin	[202]
Bacterial survival	Hemin-binding protein, TonB-dependent receptors	β-lactamase protein	[202, 203]
Bacteria adhesion and invasion	Adhesin/invasin, OmpA	Plasma binding proteins, staphopain A	[202]
Immune evasion	Cytotoxic necrotizing factor 1, UspA1/A2	Coagulation factors, antibody degradation and sequestering factors, complement inhibition factors	[46, 202]
Host-cell modulation	Cytolysin A, VacA toxin, CNF1, heat-liable enterotoxin, shigatoxin, Cif, flagellin, α-haemolysin	Proteolysin, β2 toxin	[202]
Killing competing bacteria	Endopeptidase L5, murein hydrolase (Mtl, Slt), peptidoglycan hydrolase	N-aetylmuramoyl-L-alanine amindase	[202]
Biogenesis	a. Loss or relocation of covalent linkages between the OM and the underlying peptidoglycan layer b. Accumulation of peptidoglycan fragments in the outer leaflet of the OM c. Misfolded proteins in periplasmic space exerting turgor pressure on OM d. Enrichment of species-specific membrane curvature-inducing molecules	Action of cell wall-degrading enzymes; endolysin, autolysin	[36]
Lipids	Glycerophopholipids, phosphatidylethanolamine, phophotidylglycerol and cardiolipin	Phosphatidylglycerol, myristic and palmitic acids	[120, 202]
Coagulation	E-selectin, P-selectin, thrombomodulin	Fibronectin binding protein, *staphylocoagulase precursor*, Vonwillebrand factor binding protein	[202]

**Table 2:** Differences and similarities in EVs deriving from eukaryotic cells and bacteria

Eukaryotic Organism	Bacteria
Spherical particles with a size range from 30 to 100 nm (exosomes), 100–1000 nm (MVs) or 500–2000 nm (apoptotic bodies) Stable at 37 °C for 24 h. Sensitive to high temperature but stable in the frozen and freeze-dried states [204]	Spherical particles with a size range from 10 to 400 nm. The maximum size is smaller than eukaryotic EVs due to smaller sized bacterial cells Stable at 37 °C for 24 h. Greater tolerance to hot temperatures [205]. Stable in the frozen and freeze-dried states
Exosomes are commonly enriched in endosome-associated proteins	Mainly composed of proteins and phospholipids of the outer membrane
Exosomes and MVs are released by healthy and damaged cells. Apoptotic bodies are released by dying cells on an apoptotic pathway	All Gram-negative bacteria produce outer membrane vesicles (OMVs) and possibly also all Gram-positive bacteria. Gram-negative bacteria can produce specific vesicles with a double layer using both the outer and inner membranes
Originates in the plasma membrane except exosomes, which are made by the endocytic pathway	Bacteria Gram-negative and Gram-positive have a different mechanism of vesicle formation due to their distinct membrane structure, which originates in the membrane
They are released from cells by a variety of mechanisms depending on their mode of biogenesis and they are not released homogeneously by the membrane	Production is not uniformly distributed along the bacteria surface but there are “hot spots”
High heterogeneity in the composition of the surface and the interior	High heterogeneity in the composition of the surface and the interior
There are universal markers such as CD40 for microvesicles or flotillin for exosomes	There are no universal markers for their identification due to their diversity
EVs can contain different RNAs such as miRNA or mRNA but it is unusual for them to carry DNA	EVs can contain genetic material and participate in horizontal gene transfer
Harmful cells such as tumor cells present EVs with specific and useful contents for their survival	In pathogenic bacteria, specific molecules have been found such as adhesins, toxins and/or immunomodulatory compounds as cargo of OMVs
The main function is intercellular communication, except for apoptotic bodies, which facilitates phagocytosis	They are more relevant as a mechanism to carry away toxic compounds for bacteria than in eukaryotic cells
Production depends on the cell type and its physiology state	Their production increases as a response to environmental stress
A non-spontaneous biological process	A non-spontaneous biological process

The production and secretion of bacterial EVs are influenced by the expression and regulation of bacterial genes, as well as the growth environment of bacteria [51, 52]. Bacterial genetic performance, such as bacterial adhesion, reproduction, and resistance to digestive enzymes and antibiotics, is closely related to the EVs secreted by bacteria [52, 53]. The production and secretion of EVs by bacteria are also affected by host age, dietary habits, antibiotic use, GI function, immune response, stress response, intestinal physical and chemical environment, and intestinal microbial composition [54–61]. Studies have found that the more vigorous the cell metabolism and the more stimulation the cell receives, the more EVs are secreted [30, 62, 63].

EVs can be categorized into different subtypes based on their pathogenesis and morphological structure. Each subtype represents different physiological or pathological states of cells and has different purposes and functions. Similar to eukaryotic EVs, bacterial EVs can be classified into exosomes, microvesicles, apoptotic bodies, and oncosomes. There may be additional subtypes of bacterial EVs that have yet to be discovered, and further research into the mechanisms of their occurrence can reveal different physiological states of bacteria and uncover a wider range of functions of EVs [8, 27, 40, 62].

## Components of bacterial EVs

Bacterial EVs are nanoscale spherical transporters that are composed of a phospholipid bilayer membrane and have a diameter ranging from 20 to 250 nm. The membrane envelops nucleic acids, proteins, lipids, and other substances, while specific lipopolysaccharides and outer membrane proteins are embedded in the outer layer of the membrane. The asymmetry and fluidity of the membrane structure are responsible for the specific structure and function of the membrane surface [9, 64, 65], which is influenced by genetic and growth environments. EVs from different bacteria have different contents, with a focus on protein sorting, protein proportion, and DNA or RNA with different functions [66].

### Proteins

Proteomic analysis has identified more than 3,500 proteins associated with OMVs [67, 68]. OMVs have been found to contain a large number of outer membrane proteins, including OmpA, OmpC, and OmpF, as well as periplasmic proteins, such as AcrA and alkaline phosphatase [67, 68]. Additionally, OMVs contain various adhesins and exotoxins [69]. The majority of OMV proteins are virulence factors that aid in the survival of bacteria by promoting bacterial growth, inhibiting competitive bacteria, evading host immunity, and resisting environmental toxins. OMVs also contain carrier proteins and channel proteins that are responsible for transport, accounting for a large proportion of OMV proteins [69]. Proteins carried by OMVs are strictly sorted based on their amino acid sequences, which contain special signal sequences known as signal peptides [70]. These signal peptides act as sorting signals that guide proteins to their target compartments. Each sorting signal is recognized by a corresponding sorting receptor. Proteins synthesized by bacterial ribosomes are transported to the inner and outer membranes, periplasm, or outside of the cell by various secretion systems. If a protein is loaded into a small vesicle, the corresponding receptor on the vesicle membrane must recognize its sorting signal signal [10, 68, 71]. The protein content of OMVs is subject to change due to alterations in gene expression and the growth environment of bacteria [70].

### Nucleic acids

Bacterial EVs have been found to contain multiple types of nucleic acids [72]. EVs can carry DNA both in the lumen and on the membrane surface. The DNA in the lumen retains its antigenicity even after treatment with DNase, distinguishing it from the membrane-bound DNA. OMVs also contain miRNAs, mRNAs, and other non-coding RNAs. Several different forms of luminal DNA have been identified in OMVs secreted by *Escherichia coli *(*E. coli*)*, Neisseria gonorrhoeae *(*N. gonorrhoeae*)*, Pseudomonas aeruginosa *(*P. aeruginosa*)*, and Haemophilus influenzae *(*H. influenzae*) [73].

OMVs have been found to contain mRNA, which can be transferred and translated after entering the host cell. Retrotransposons and other non-coding RNAs have also been reported in OMVs [74, 75]. The discovery of various nucleic acid types in OMVs highlights their importance as carriers and transmitters of genetic information, although the mechanism by which nucleic acids enter OMVs remains unclear. Similar to the intracellular transport of proteins, it is speculated that the intracellular transport of nucleic acids may involve corresponding recognition sequences through which nucleic acids are selected to enter OMVs [76, 77].

### Lipids

The basic structure of EV membranes is composed of lipids, primarily phospholipids, and lipopolysaccharides. In *E. coli* OMVs, glycerophospholipid, phosphatidylglycerol, and phosphatidylethanolamine are important lipid components that contribute to the curvature of the OMVs [78]. Lipopolysaccharide (LPS) is an endotoxin and serves as an important antigen and ligand on the membrane surface, playing a crucial role in adhesion and activating the immune response. LPS is composed of three parts: lipid A, core polysaccharide, and O antigen. Lipid A is the most toxic component, while the O antigen is exposed on the membrane surface and serves as an important antigenic determinant [79, 80].

### Role of bacterial EVs in the gut

EVs secreted by intestinal bacteria can diffuse in the intestinal microenvironment or enter the bloodstream. They are capable of recognizing specific molecules present in the environment through ligands on their membrane surface and can also be recognized and bound by specific receptors on the membrane surface of target cells. Once inside the cells, they can transmit substances and activate specific signaling pathways to transmit information [81, 82]. EVs are powerful tools that can deliver, bind, and transform substances (Fig. 2) [53, 83]. These vesicles carry various substances that have been screened by bacteria, representing the bacteria’s viability. They play a similar role to bacteria and have a significant impact on host cells, intestinal microorganisms, and the intestinal environment [12, 84].

EVs have different effects on the growth, reproduction, and colonization of bacteria of the same species. They also have both advantages and disadvantages for other bacteria and host cells. For example, they can promote the colonization of probiotics and regulate the immune response, which is beneficial to the host. However, they can also destroy the host mucosal barrier and cause inflammatory storms that are harmful to the host. The current study has identified the role of bacterial EVs in the gut, which is summarized in Table 3. This includes related studies on the role of EVs from known intestinal bacteria in the intestine [13].

**Table 3:** Studies evaluating the role of microbiota derived BEVs as modulators of intestinal homeostasis-related processes

Bacteria	Mechanism	Experimental approach	References
Gut ecology and food metabolism			
* Bacteriodes fragilis* * B. thetaiotaomicron*	Metabolism of complex carbohydrates to produce SFCAs: Expression of glycosyl-hydrolases, sulfatases, proteases Cholesterol uptake: upregulation NPC1L1 receptor Metabolites in BEVs that facilitate intestinal colonization	Proteomics of BEVs by mass spectrometry In vitro Caco-2 cell culture In silico, proteomic and metabolomic analysis	[137, 188, 206–208]
* B. thetaiotaomicron*	Assimilation of dietary Insitol-P Macrophage internalization (Sulfatases)	Biochemical characterization of InsP6-phosphatase Experimental model of colitis using genetically modified mice	[209, 210]
* Bacteroides fragilis*	Antibiotic resistance (β-lactamases)	Knockout mutant of putative β-lactamase gene	[150]
Epithelial barrier integrity			
* E. coli* Nissle 1917 ECOR63 strain	Upregulation of TJ proteins ZO-1 and claudin-14, downregulation of claudin-2 Protection against EPEC-induced damage: preservation of occludin and claudin-14 mRNA levels, redistribution of ZO1, amelioration of F-actin disorganization	In vitro Caco-2 and T-84 cell cultures: RT-qPCR, confocal microscopy In vitro Caco-2 and T-84 cell cultures infected with EPEC: RT-qPCR, confocal microscopy, paracellular permeability assays	[116, 211]
* E. coli Nissle 1917*	Upregulation TFF3 and MMP-9 mRNA	In vivo mice model of DSS-induced colitis	[117]
* Akkermansia muciniphila*	Upregulation of ZO-1, claudin 5 Upregulation of ZO-1, ccluding, claudin-1 Upregulation of ccluding, ZO-1/2, claudin-4	In vivo high-fat diet (HFD)-induced diabetic mice model, and Caco-2 cell culture In vivo HFD-induced obesity mice model In vitro Caco-2 cells challenged with LPS	[212–214]
Gut immunity: modulation of inflammatory responses through the intestinal epithelium			
* E. coli Nissle 1917* ECOR12 strain	Upregulation of IL-6, IL-8, TNF-α, IL-10, MIP1α Upregulation of IL-22 and β-defensin Downregulation of IL-12 Activation of NOD-1 / NF-κB pathway	In vitro Caco-2/PBMCs cell coculture model Ex vivo model of colonic explants Caco-2 cells: NOD1 silencing—RIP2 kinase inhibition	[215, 216]
* E. coli Nissle 1917*	Upregulation of IL-10; downregulation of IL-1β, TNF-α, IL-6, IL-12, IL-17, iNOS and COX-2 in colonic tissue	In vivo mice model of DSS-induced colitis	[117]
* Lactobacillus kefir* * L. kefiranofaciens* * L. kefirgranum*	Downregulation of IL-8 Counteract oxidative stress by decreasing myeloperoxidase serum levels	Caco-2 cells challenged with TNF-α In vivo mice model of TNBS-induced IBD	[217]
Gut immunity: modulation of DCs and derived T cell responses			
* Bacteroides fragilis*	Induction Treg cells (CD4 + CD25 + FOXP3 +) and IL-10 production through a mechanism that involves TLR2 Activation of autophagy. Induction of Treg cells and IL-10 production depends on functional ATG16L1 and NOD2	In vivo mice model of TNBs-induced colitis In vitro bone marrow-derived DCs culture BEVs from wild-type and PSA deficient strains Bone-marrow derived DCs from wild type, ATG16L1- and NOD2 deficient mice In vitro cocultures of BMDCs with CD4^+^T cells In vivo mice model DNBS-induced colitis	[94, 96]
* Bacteroides vulgatus mpk*	Induction of DC tolerance via TLR2 and TLR4 Upregulation of co-stimulatory molecules including MHC-II, CD40, CD80 and CD86 in CD11c^+^ cells	In vitro bone marrow-derived DCs culture TLR4/TLR2 knockout mice model	[218]
* Lactobacillus rhamnosus* JB-1	Increased production of IL-10 and regulatory (CD4^+^CD25^+^FOXP3^+^) T cells	In vitro bone marrow-derived DCs culture In vivo mice model	[219]
* Lactobacillus sakei*	Enhance IgA expression	Ex vivo model of murine Peyer's patches	[220]
* Bifidobacterium bifidum* LMG13195	Promote differentiation to regulatory T cells (CD4^+^CD25^+^FOXP3^+^) and IL-10 secretion	In vitro model of monocyte-derived DCs co-cultivated with CD4^+^ T cells	[221]
* Bifidobacterium longum*	Apoptosis of bone-marrow-derived mast cells through ESBP vesicular protein	In vivo mouse model of allergen-induced food allergy	[222]
* E. coli Nissle* 1917 Commensal *E. coli*	Upregulation of driver Th cytokines by DCs in a strain-specific manner Differential induction of Th1, Th2, Th17/Th22 and T regulatory responses Regulation of key miRNAs in immunity (miR-155, miR-146a/b and miR-let7i) Differential modulation of miRNAs involved in tolerogenic responses (miR-125a/99b/let7e, miR-125b, miR-24)	In vitro model of monocyte-derived DCs co-cultivated with CD4^+^ T cells In vitro model of monocyte-derived DCs: RNA seq approaches to identify differential expressed miRNAs	[223, 224]

### Host cells

#### Intestinal immune cells

**Fig. 3:**
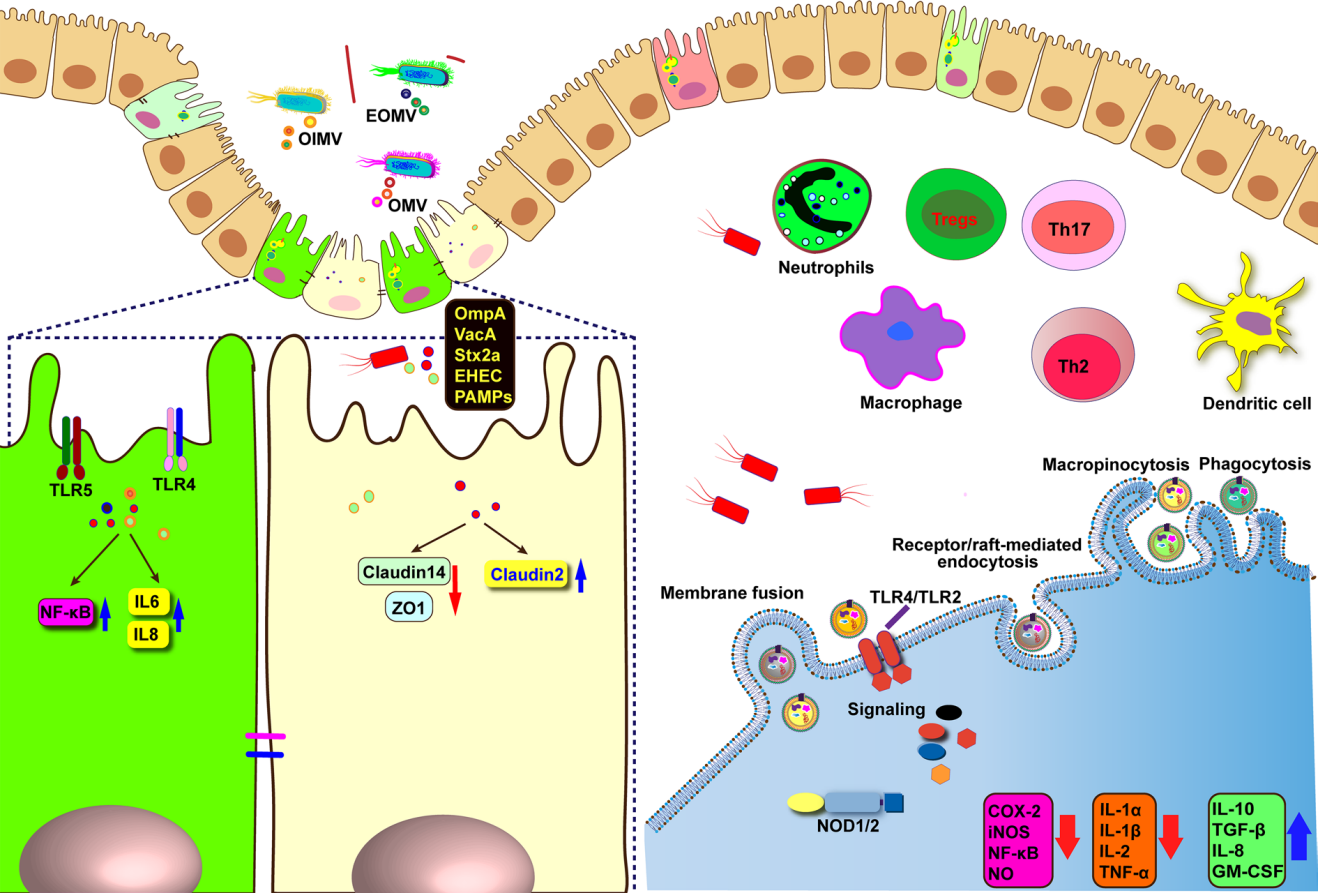
Bacterial outer membrane vesicles play an important role in bacterial interactions with human intestinal epithelial cells and intestinal immune cells

EVs recognize and stimulate immune cells through pathogen-associated molecular patterns (PAMPs), mainly including specific antigens, such as LPS, peptidoglycan on the surface of the membrane, and DNA in the cell. They combine with pattern recognition receptors (PRRs) for target recognition [85, 86]. PRRs associated with bacterial EVs include Toll-like receptors (TLRs) on the cell surface, such as TLR4 activated by LPS, and NOD-like receptors (NLRs) in cells, such as NOD1 and NOD2, activated by peptidoglycan components. Cysteine-containing aspartic proteolytic enzymes (Caspases), such as Caspase-11, which act as intracellular receptors for LPS, mediate the activation of an intracellular inflammatory pathway in OMVs (Fig. 3) [87].

When bacterial EVs recognize and bind to immune cells, whether they are beneficial to host immune regulation depends on the source of bacteria and the substances they carry [88–90]. Firstly, LPS and peptidoglycan on the surface can stimulate intestinal immune cells, trigger an inflammatory response, and maintain normal intestinal immune function. However, they can also trigger immunosuppression, an excessive immune response, or induce immune tolerance, which may lead to bacterial invasion or infection of the host and enable the bacteria to evade the host's immune response [91, 92]. Secondly, sDNA or DNA antigenicity or specific virulence factors in EVs can invade immune cells by endocytosis or endocytosis, affect the expression of inflammatory factors in immune cells, induce apoptosis of immune cells, and create conditions for bacterial invasion or infection [87, 93].

EVs derived from probiotics have mostly been shown to have beneficial effects on host immune regulation. For example, OMVs secreted by *Bacteroides fragilis *(*B. fragilis*) carry polysaccharide A (PSA) and are delivered to intestinal dendritic cells, which can induce CD4 + regulatory T cells (Tregs) to produce IL-10, down-regulating inflammatory responses and effectively ameliorating DSS-induced colitis of the colon [94–96]. Similarly, OMVs secreted by *Akkermansia muciniphila *(*A. muciniphila*) have been shown to significantly down-regulate DSS-induced colitis in mice and play an important role in regulating inflammatory immune response and maintaining the intestinal immune barrier [97]. *Lactobacillus paracasei *(*L. paracasei*) is a probiotic with anti-inflammatory properties, and in vitro studies have shown that EVs from *L. paracasei* (LpEVs) can down-regulate the expression of proinflammatory cytokines, such as IL-1α, IL-1β, IL-2, and TNF-α, and up-regulate the expression of anti-inflammatory cytokines, such as IL-10 and TGF-β. Additionally, LpEVs can inhibit the activation of inflammatory proteins, such as COX-2, iNOS, NF-κB, and nitric oxide (NO), in signal transduction pathways and significantly inhibit the inflammatory response of human colon adenocarcinoma HT-29 cells induced by LPS. Animal experiments have also demonstrated that oral administration of LpEVs can significantly prevent the reduction in body weight, colon length, and disease activity index (DAI), thus attenuating clinical signs in DSS-induced mice [98].

According to Kim et al. [99], EVs derived from pathogenic bacteria often result in host immune abnormalities [99]. For example, *E. coli* OMVs transmit virulence factors to host intestinal macrophages, which up-regulate the expression of proinflammatory cytokines, such as IL-6 and TNF-α. This leads to systemic inflammatory response syndrome (SIRS) and sepsis. Additionally, the *heat-labile enterotoxin *(*LT*) on the surface of enterotoxigenic *E. coli* OMVs interacts directly with host cells through PRRs, activating proinflammatory signaling pathways and chemokines expressed by host cells and ultimately causing inflammatory responses [100–102]. Furthermore, *Vibrio cholerae *(*V. cholerae*)* O395* OMVs are taken up by intestinal epithelial cells through caveolin-mediated endocytosis of outer membrane porins (OmpU and OmpT). This induces the expression of proinflammatory cytokines (such as IL-8 and GM-CSF) and chemokines (such as CCL2 and CCL20), leading to the polarization of T cells to Th2/Th17 and causing an inflammatory response [103, 104]. In the case of *Helicobacter pylori *(*H. pylori*), OMVs bind to human monocytes and deliver virulence factors, such as vacuolating cytotoxin (VacA), strongly up-regulating the expression of inflammatory cytokines, such as IL-6 and IL-10. This inhibits the proliferation of CD4 + T cells and induces T cell apoptosis [105]. Flagellated bacteria, such as *Salmonella typhimurium *(*S. typhimurium*) and *Pseudomonas aeruginosa*, release OMVs that cause strong NLRC4-mediated caspase-1 activation and IL-1β secretion in macrophages in an endocytosis-dependent manner, promoting an inflammatory response [106]. Besides, *Acinetobacter baumannii *(*A. baumannii*) OMVs containing the virulence factor OmpA target mitochondria in mice and disrupt the mitochondrial morphology of mouse macrophages [107, 108].

#### Intestinal epithelial cells

Bacterial EVs are capable of being recognized by intestinal epithelial cells and can enter them through various mechanisms, including macropinocytosis and clathrin-mediated endocytosis, in order to transmit substances or signals [10]. Depending on the components of OMVs, they may provide nutrients and digestive enzymes that are necessary for metabolism and help repair the intestinal epithelial barrier. However, they may also have harmful effects, such as damaging the intestinal epithelial cells, destroying the intestinal epithelial barrier, and inducing intestinal epithelial cell death, including apoptosis, necrosis, autophagy, and other harmful effects [99, 109–114].

Bacteroides OMVs have been discovered to carry human therapeutic keratinocyte growth factor 2 (KGF-2), which promotes the repair of intestinal epithelial cells in DSS-induced colitis in mice after oral administration [115]. Other studies have demonstrated that the oral administration of EVs secreted by *E. coli Nissle 1917 *(*EcN*), *L. paracasei*, and *B. fragilis* can promote the repair of intestinal epithelial cells and the intestinal mucosal barrier in DSS model mice, significantly improving the inflammatory response [98, 116, 117]. OMVs secreted by *EcN* and a human *E. coli* strain containing the tcpC gene (ECOR 63) can up-regulate ZO1 and claudin14 while down-regulating claudin2 in intestinal epithelial cells, which helps enhance the tight junction between intestinal epithelial cells and reduce intestinal permeability (Fig. 3) [116].

Studies have demonstrated that OMVs secreted by various enterobacteria contain OMV-related virulence factors that can trigger the death of human intestinal epithelial cells, as well as the release of inflammatory factors [118]. The outbreak strain *E. coli* O104: H4 has been found to release OMVs carrying virulence factors, including *Shiga toxin *(*Stx2a*), which enters into intestinal epithelial cells, targets the mitochondria, and induces the release of cytochrome C. This, in turn, activates the caspase-9 and caspase-3 pathways, leading to the formation of apoptotic bodies and the apoptosis of intestinal epithelial cells [113]. [113]. Enterohemorrhagic *E. Coli* (EHEC) releases OMVs containing the virulence factor EHEC hemolysin (EHEC-Hly), which is taken up into lysosomes through endocytosis by intestinal epithelial cells. EHEC-Hly then escapes from lysosomes and targets mitochondria, causing a decrease in mitochondrial transmembrane potential and the release of cytochrome C. This induces the formation of apoptotic bodies and triggers apoptosis of intestinal epithelial cells by activating caspase-9 conduction channels [119, 120].

In Crohn’s disease (CD), the endoplasmic reticulum-localized stress response protein (ER-localized stress response protein, Gp96) is overexpressed on the surface of ileal epithelial cells. PAMP molecules carried by OMVs derived from Adherent-invasive *E. coli* (AIEC) recognize and bind to Gp96 receptors on the surface of intestinal epithelial cells, promoting AIEC invasion and mediating the destruction of intestinal mucosal epithelial cells in CD) [110, 121]. *Fusobacterium nucleatum *(*F. nucleatum*) secretes EVs containing a variety of virulence factors that promote M1 polarization of macrophages, leading to oxidative stress injury of intestinal epithelial cells. These EVs also activate receptor-interacting protein kinase 1 (RIPK1) and receptor-interacting protein kinase 3 (RIPK3), ultimately leading to the activation of caspase-3-related signaling pathways. This promotes programmed cell necrosis of intestinal epithelial cells and destruction of the intestinal epithelial barrier [122].

#### GI tumor cells

Research on the effect of bacterial EVs on GI tumor cells is still in its early stages. However, current studies have found that some bacterial EVs can invade host cells, causing chronic inflammatory responses, damaging genetic material, and increasing the risk of host cell canceration [123]. Bacterial EVs have also been found to be capable of crossing physiological barriers and selectively accumulating near tumor cells, potentially altering the tumor microenvironment (TME) [124, 125].

Bacterial EVs use enhanced permeation and retention (EPR) effects and EPMP antigen molecules to induce tumor immune responses. Whether EVs can recognize introduced cells in tumor tissues and induce targeted tumor cell death requires further research. Nevertheless, modifying bacterial EVs to load chemotherapeutic drugs or anti-tumor components may be a new direction for anti-tumor therapy in the future [126, 127]. Animal experiments have shown that a mixture of *Bifidobacterium lactis (B. lactis)* and *Lactobacillus rhamnosus *(*L. rhamnosus*) can improve the level of intestinal butyrate, reduce the proliferation of cancerous cells, and decrease the activity of enzymes related to rectal cancer occurrence, thereby reducing the colon cancer morbidity of rats [128].

In mouse models, several intestinal microflorae have been found to promote colorectal cancer (CRC). For example, Enterotoxigenic *B. fragilis* (ETBF) and *E. coli* both carry PKS islands to promote toxin production, while *F. nucleatum* promotes CRC growth by stimulating inflammation and activating the β-catenin pathway [128, 129].

#### Exo-intestinal somatic cells

Some bacterial EVs can be absorbed into the blood and circulate to extraintestinal target cells, such as vascular endothelial cells, blood cells, and central neurons, causing diseases in cardiovascular, metabolic, and central nervous system (CNS) diseases [130]. Additionally, bacterial EVs can alter intestinal microecology and cause multi-system diseases. For instance, OMVs secreted by *Bacteroides thetaiotaomicron *(*Bt*) can be endocytosed and captured by intestinal epithelial cells through paracellular pathways and migrate to the submucosal and circulatory systems [131, 132].

### Intestinal microorganisms

The composition of the intestinal microbiota is not solely determined by competition among microorganisms but also by their ability to sense and adapt to the intestinal microenvironment. The viability and defense mechanisms of microorganisms determine their ability to colonize and thrive in the gut. EVs derived from probiotic bacteria can support the growth and colonization of beneficial microorganisms while inhibiting the growth and colonization of foreign microorganisms, which are typically pathogenic or opportunistic. These EVs are crucial for maintaining the stability of the intestinal microbiota. When the microbiota becomes dysbiotic, pathogenic EVs can inhibit the growth and colonization of probiotics, alter the structure of the microbiota, and disrupt the homeostasis of the intestinal microecology.

Bacteria secrete EVs to deliver essential nutrients, functional genes, and enzymes with varying functions to the same flora. This enables the bacteria to enhance their viability and survive in the changing microenvironment of the intestine [133, 134]. *Bifidobacterial* EVs contain mucin-binding proteins that promote the colonization of *bifidobacteria* in the intestinal mucosa [135]. Similarly, *Bacteroides ovatus *(*B. ovatus*) OMVs carry inulin-degrading enzymes, which can degrade inulin and produce nutrients to support the growth of other Bacteroides species that cannot utilize inulin [136]. Proteomic analysis has revealed that OMVs of *B. fragilis* and *Bacteroides thetaiotaomicron *(*B. thetaiotaomicron*) selectively package a large number of carbohydrate hydrolysis and proteolytic enzymes that can digest and absorb various polysaccharides. These OMVs provide nutrients for bacteria that cannot decompose polysaccharides and help maintain the stability of the intestinal microecology [136, 137].

Furthermore, OMVs secreted by normal *H. pylori* have been found to promote biofilm formation in *non-membranous H. pylori* strains, demonstrating their ability to enhance biofilm formation [136].

EVs secreted by bacteria have the ability to transfer virulence factors to competitive flora and host cells. This can result in structural damage or dysfunction of competitive flora and host cells and even lead to cell death [138]. However, host cells and competing flora have corresponding defense mechanisms to resist the destructive effects of virulence factors, and they are constantly fighting and evolving, with the winner surviving and the loser migrating [139].

For instance, OMVs isolated from the foodborne pathogen E. coli O157:H7 have been shown to transfer virulence factors and other genetic material to recipient bacteria, such as *E. coli* JM109 or *Salmonella enterica* serovar irritable bowel [140]. This transfer enhances the cytotoxicity and defense ability of recipient bacteria [141]. *Burkholderia cepacia *(*B. cepacia*) have been shown to have strong antibacterial activity against *A. baumannii*, *S. aureus*, multidrug-resistant *A. baumannii*, methicillin-resistant *S. aureus*, and *fungal pathogens*. Further chemical analysis of OMVs derived from *Burkholderia tylanica* reveals that they carry peptidoglycan hydrolase and proteolytic enzymes, as well as antibacterial molecules, such as 4-hydroxy-2-alkylquinoline and rhamnolipid compounds. These molecules can affect ionophores, iron chelation, immunomodulation, and intercellular communication [142].

In addition, EVs secreted by bacteria can also carry defense factors, including virulence factors produced by various bacteria, toxic molecules in the environment, phage invasion, and host immune response. For instance, *P. aeruginosa* OMVs carry extracellular DNA, which not only mediates evasion of the host immune response but also promotes resistance to aminoglycoside antimicrobial agents [143]. Moreover, bacterial EVs are the primary means of HGT for spreading antibiotic resistance genes (ARGs), leading to reduced therapeutic efficiency of antibiotics and posing a serious threat to human health [144–146]. EVs can also transfer β-lactam resistance genes into and out of bacterial species, enhancing resistance to β-lactam antibiotics in many bacteria [147, 148]. Additionally, the number of active *bacteriophages* is significantly reduced by about 90% after co-incubation of *T4 bacteriophage* and EVs, indicating that EVs can combine irreversibly with *T4 bacteriophages* and thus significantly reduce their numbers in the environment. This, in turn, reduces the chance of bacterial infection by bacteriophages, as measured by the number of plaque-forming units [149].

### Intestinal environment

The contents of the intestine primarily include chyme, mucus, and bacteria. Mucus is the exocrine fluid secreted by host cells, while the chyme is the digested food by the GI tract and serves as a shared resource for both host and bacteria. Each bacterium competes for high-quality resources and degrades harmful substances. Bacteria secrete EVs that carry digestive enzymes and transformed nutrients, which integrate into the intestinal chemical environment, digest chyme, provide nutrients, and transform harmful substances to bacteria, such as immune antibodies, antibiotics, toxic molecules, and phages, to improve the chemical environment for bacteria. Additionally, OMVs contain numerous enzymes that can degrade biological macromolecules. Therefore, when macromolecular substances are present in the living environment of bacteria, the release of OMVs can degrade them, enabling the bacteria to absorb and utilize these nutrients effectively [5, 27]. Secondly, OMVs have the ability to adsorb and bind antibiotics, thereby reducing their concentration, carrying antibiotic hydrolase, degrading antibiotics, and horizontally transferring ARGs to enhance the antibiotic resistance of bacteria [5, 7]. For instance, EVs released from *S. Aureus* under the stress of ampicillin contain a large number of proteases that can degrade β-lactam antibiotics and neutralize them in the environment [58]. Furthermore, EVs secreted by *Bacteroides* spp. containing β-lactamase can hydrolyze β-lactam antibiotics, reduce the concentration of antibiotics in the intestinal microenvironment, and improve the survival rate of intestinal symbiotic bacteria [150]. In a study, it has been reported that polymyxin treatment can induce sewage bacterial communities to produce a large number of EVs in the real environment, and these EVs can potentially reduce the concentration of antibiotics in water [151]. It has also been observed that polymyxin B and colistin, which are polypeptide antibiotics, can induce *E. coli* to release EVs that can adsorb antimicrobial peptides, thus eliminating the killing effect of these peptides on bacteria, possibly due to the binding of LPS carried by EVs to antimicrobial peptides [149, 152, 153].

Although excreted feces still contain a significant number of intestinal bacterial EVs, these EVs can remain stable and active in vitro. They carry a diverse range of enzymes and active molecules that degrade intestinal chyme, which can alter the characteristics of intestinal contents or feces, stimulate the mechanical movement of the intestine, and influence defecation patterns. In general, probiotics and their EVs can stimulate GI motility, improve stool characteristics, and promote regular bowel movements. In cases of dysbiosis, pathogenic bacterial EVs can weaken GI motility, result in dry stools, and cause constipation or diarrhea.

## Potential application of bacterial EVs in GI

**Fig. 4:**
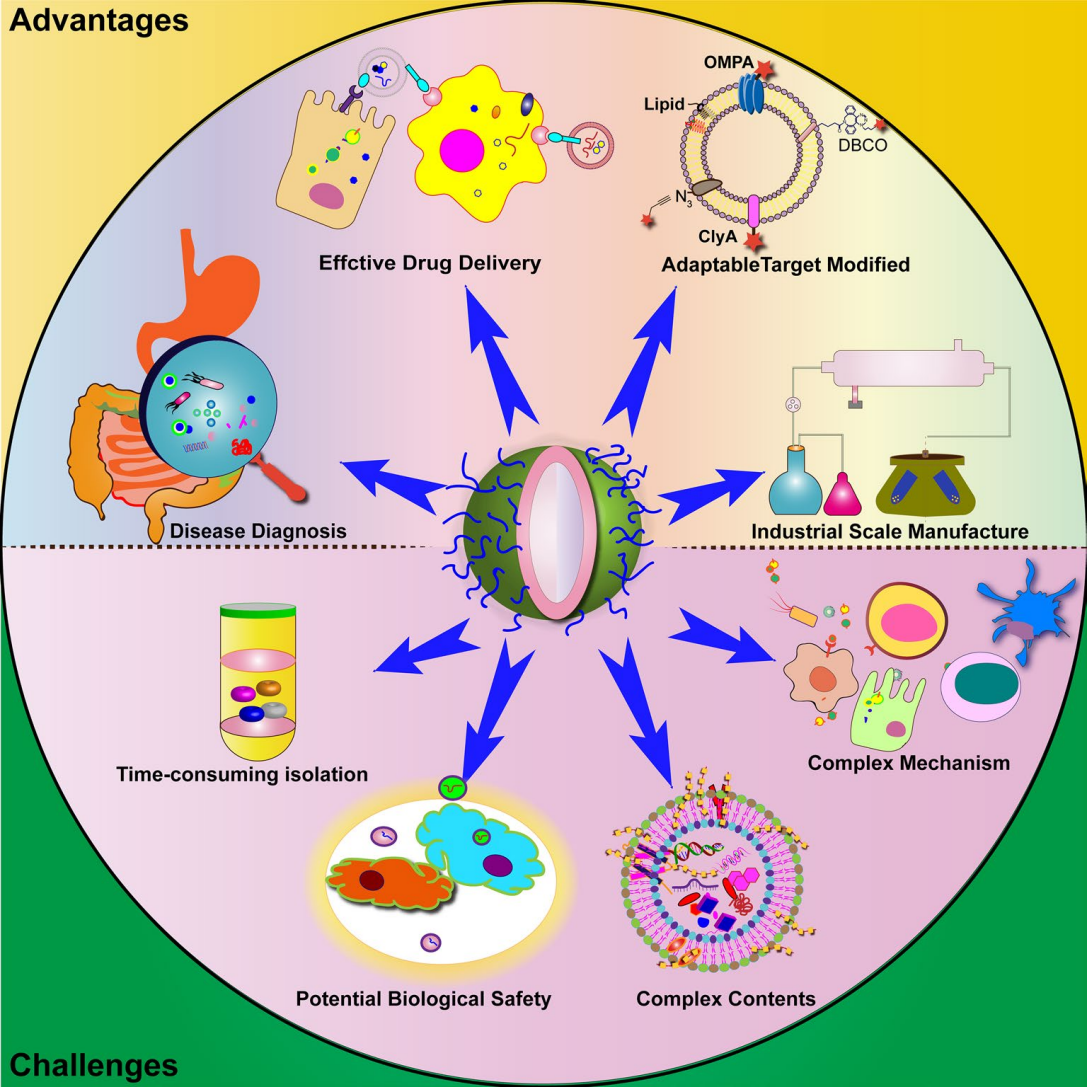
The advantages and challenges of bacterial-derived outer membrane vesicles. OMV possess inherent adjuvanticity

Bacterial EVs play a wide range of roles in the GI system. Although still in the basic research stage, bacterial EVs exhibit greater diversity and functionality than somatic EVs. They possess strong immunogenicity and can be conveniently detected in feces, urine, blood, and other bodily fluids. Obtaining bacterial EVs is relatively easy, thanks to mature bacterial culture and strain isolation technologies. Moreover, ideal EVs can be obtained through regulation. Bacterial EVs hold immense potential in disease diagnosis, vaccine or immune adjuvant development, intestinal microecology maintenance, and drug delivery. Currently, research technology presents the biggest obstacle to realizing this potential [154, 155]. However, as EV research technology matures, bacterial EVs will likely demonstrate even greater application potential (Fig. 4).

### Diagnostic biomarkers

By detecting changes in bacterial EVs in bodily fluids, such as blood, feces, urine, and others, we can gain insight into the composition of the gut microbiota and identify imbalances in a timely manner. These bacterial EVs carry a large number of specific biological macromolecules that serve as the basis for the directional recognition and immunogenicity of bacterial EVs and are also key molecules in the development of intestinal and extraintestinal diseases. Discovering new biomarkers for the diagnosis of GI and extraintestinal diseases, as well as monitoring disease progression and treatment efficacy, is of great importance [156, 157]. For example, the levels of antibacterial EV antibodies in the blood of patients with bronchial asthma, chronic obstructive pulmonary disease (COPD), and lung cancer are found to be significantly higher than those of healthy controls, indicating that measuring EVs in these patients may serve as a diagnostic biomarker [158]. It is believed that in the near future, specific components of bacterial EVs in human bodily fluids can be identified to aid in the diagnosis of intestinal diseases.

### Therapeutics

Bacterial EVs have a significant impact on host cells and intestinal microorganisms, rendering them a potential therapeutic tool for regulating gut microbiota imbalances and improving immune function. By modulating the balance of the gut microbiota and maintaining intestinal immune barriers, bacterial EVs can indirectly improve the intestinal microenvironment, protect the intestinal epithelial and mucus barriers, and enhance intestinal digestion, absorption, and defecation functions.

#### Regulation of flora disorder

Dysbiosis is considered to be the primary factor or a concurrent change in the development of various diseases, and the regulation of gut microbiota is becoming a key aspect of disease treatment. Traditionally, antibiotics, oral probiotics, and other methods have been used to control pathogenic bacteria. However, in some patients, these methods are ineffective and may even result in antibiotic-associated diarrhea. Fecal microbiota transplantation (FMT) has been successful in treating refractory diarrhea [159]. Compared to the direct administration of microbial agents, using bacterial EVs as a therapeutic approach has several advantages, such as the ability to cross the intestinal barrier, low toxicity, high plasticity, and specificity [155]. The future direction is to use EVs to control bacteria and maintain the homeostasis of gut microbiota through EVs, which can be more accurate and effective. The introduction of EVs from probiotics can not only inhibit the reproduction and colonization of pathogenic bacteria but also provide nutrients for normal gut microbiota and resist harmful substances, such as reactive oxygen species, antibiotics, and antimicrobial peptides [53].

#### Regulation of immune function

Bacterial EVs are known to carry specific immunogens and PAMP molecules that are related to the parent bacteria. They have strong immunogenicity and immune cell recognition ability, which makes them suitable for developing bacterial outer membrane vesicle vaccines or immune adjuvants [85, 93, 160]. For example, some countries, such as Cuba, Norway, New Zealand, and the Netherlands, have developed monovalent vaccines against local prevalent strains of group *B meningococcal *(*MenB*) using detergent-extracted outer membrane vesicles (dOMVs) as one of the primary antigens [161–164]. The process of dOMV detergent extraction reduces LPS/lipoprotein content and hence OMV endotoxicity. It can also induce the production of bactericidal antibodies against cell-surface outer-membrane proteins (OMPs) [165]. Based on this, a polyvalent *MenB* vaccine (MenB-4C) was developed by Novartis (China) Biomedical Research Co. in 2013. This vaccine contains dOMV and three recombinant proteins, and it can cover 66–91% of MenB strains worldwide [165–167]. EVs are known to carry a significant amount of LPS, which not only acts as a potent immunogen but also generates a strong heat source. LPS is a key component of virulence factors, which can cause damage to host cells, trigger immune cell inflammation, and even lead to death [168, 169]. However, lysozyme can bind strongly to LPS, and the complex formed by lysozyme can help to inhibit inflammatory responses. As a result, OMVs can be detoxified by lysozyme [170, 171].

Currently, OMVs are treated with detergents, such as sodium deoxycholate, to remove a large amount of LPS. However, this process can also cause the loss of some immunogenic lipoproteins [172]. To address this issue, researchers have introduced OMVs of *Neisseria meningitidis *(*Nm*) LpxL 1 mutant gene, which converts lipid A from six fatty acyl chains to five fatty acyl chains. This modification has been shown to reduce pyrogen toxicity to the host while retaining moderate immunogenicity of LPS, thus ensuring the effectiveness and safety of vaccine products. This approach is still in the clinical research Stage [173, 174]. In other studies, OMV vaccines have been developed against *Mycobacterium tuberculosis *(*M. tuberculosis*) and Staphylococcus aureus, which produce protective cellular and humoral immune responses in mice [175, 176]. Furthermore, vaccination with *S. aureus* OMVs has been shown to protect against active *S. aureus* infection [177]. Currently, OMV vaccines against other bacteria, including *H. pylori*, *V. cholerae*, and *Klebsiella pneumoniae *(*K. pneumoniae*), and genetically engineered Bacteroides OMV vaccines are in the late stages of research and development [115, 178, 179].

Another promising approach in vaccine development involves mimicking the mechanism of OMVs and incorporating specific immunogens of pathogenic bacteria into the ideal OMV vector [180–182]. For example, non-pathogenic *E. coli* OMVs are engineered to express *Streptococcus pneumoniae *(*S. pneumoniae*) surface glycans. These OMVs can generate immune responses comparable to commercially available Streptococcus pneumoniae vaccines [183].

To address bacterial resistance, the development of bacterial vaccines should focus on innovative solutions. In one study, bovine serum albumin (BSA) is encapsulated with OMVs to create a BSA-OMV nano-vaccine. This approach significantly improves the survival rate of mice infected with a lethal dose of carbapenem-resistant *Klebsiella pneumoniae *(*CRKP*) [184].

### Drug delivery

Bacterial EVs possess stability and targetability, allowing them to recognize specific molecules and cells with an EPR effect. As non-toxic drug carriers with good human compatibility, bacterial EVs can improve the efficacy of drugs [155, 185, 186]. To prepare bacterial EVs for drug delivery, screening of suitable bacterial EVs should be done first and then transformed and modified to recognize and load drugs or bioactive substances. Specific PAMP molecules should be present on the surface of vector EVs for directionally recognizing target cells and introducing drugs and bioactive substances. Synthetic nanomaterial carriers lack the ability to replicate the surface features of vesicles, lack intercellular interaction, and lack targeting recognition ability [187]. OMVs derived from *B. fragilis* have the potential to be used as drug carriers for the treatment of intestinal diseases [188]. EVs secreted by Bacillus subtilis can transport across the GI epithelium, which is useful for food, nutrition, health care products, and clinical treatment [189]. [190]. Combining OMVs with miRNA can be used to treat cancer, such as intestinal cancer, where OMVs extracted from intestinal bacteria can encapsulate anti-tumor miRNA and be delivered orally to cancer tissues in the GI tract [190]. The combination of OMVs and miRNA can effectively inhibit metastatic tumor cells. *E. coli* OMVs encapsulating mediating pore silica and combined with 5-fluorouracil can enhance the drug concentration at a colon part and release the drug centrally in the TME, resulting in reduced systemic adverse reactions and improved treatment of CRC [191]. Levofloxacin-loaded *A. baumannii* OMVs can effectively invade *E. coli*, *P. aeruginosa*, and *A. baumannii*, kill *E. coli*, and produce good therapeutic effects in a mouse intestinal *E. coli* infection model [192]. Exogenous siRNA carried by cellular EVs can inhibit oncogene expression by targeting mRNA. Synthetic nanocarriers have made some progress in silencing oncogene expression with exogenous siRNA, and bacterial EVs are under research for this purpose [123, 193]. OMVs can also be adapted for genetic engineering and chemical engineering methods similar to eukaryotic exosomes for targeting delivery [194–199]. Targeting intestinal tissue delivery of OMVs is particularly meaningful for treating IBD [200].

## Conclusions

The intestinal flora, which has coexisted with humans for hundreds of millions of years, is closely intertwined with human health and disease. The relationship between humans and microorganisms will continue to shape human health and disease in the future. Therefore, we must expand our research and knowledge of microorganisms and comprehend their evolution and variations, as this is the path towards ensuring human survival indefinitely.

EVs derived from somatic cells have shown promising results in treating GI diseases. In particular, EVs derived from intestinal epithelial cells, macrophages, and mesenchymal stem cells are currently undergoing clinical trials. However, bacterial EVs have even more diverse types and functions, stronger immunogenicity, and greater plasticity than human-derived EVs. OMVs are natural immune adjuvants that play a critical role in vaccine production, infection prevention and control, tumor treatment, and drug delivery. Bacterial EVs offer several advantages in maintaining intestinal microecology, regulating immunity, and serving as drug carriers, making them a promising candidate for a broad range of applications (Fig. 4).

However, there are several challenges that need to be addressed: (1) The biological mechanisms and structural components of bacterial EVs are not fully understood, necessitating further research. (2) Identifying OMV components is complex, and the functions of various components need to be determined. (3) Techniques for extracting, identifying, shaping, and preserving EVs are not yet ideal and can limit the development of related clinical applications. (4) Further research is required to mitigate the adverse effects of reducing the activity and toxicity of OMV surface antigens.

To overcome these obstacles, the development of small-molecule inhibitors or novel strains with bacterial toxin activity knocked out on the surface of OMV may be an effective strategy for reducing the immunogenicity of OMVs. It is reasonable to believe that with the advancement of technology, these challenges will be overcome, and OMV-based nanotechnology will develop into a powerful toolkit for intestinal targeted delivery, GI disease diagnosis, treatment, and other related fields.

## Data Availability

All relevant data were included in the paper.

## Abbreviations

IBD: Inflammatory bowel disease
DMAIDs: Disease-modifying anti-IBD drugs
PDENs: Plant-derived exosome-like nanoparticles
UC: Ulcerative colitis
CD: Crohn’s disease
DDSs: Drug delivery systems
EXPO: Exocyst-positive organelle
MVBs: Multivesicular bodies, ESCRT, endosomal sorting complex required for transport
PA: Phosphatidic acid
PC: Phosphatidylcholines
DGDG: Digalactosyldiacylglycerol
MGDG: Monogalactosyldiacylglycerol
TEM: Transmission electron microscopy
SEM: Scanning electron microscopy
AFM: Atomic force microscopy
DLS: Dynamic light scattering
PE: Phosphatidylethanolamine
mTOR: Mammalian target of rapamycin
MAPK: Mitogen-activated protein kinase
mRNA: MicroRNA
sRNA: Small RNA
PEG: Polyethylene glycol
GDENs: Grape-derived exosome-like nanoparticles
DSS: Dextran sulphate sodium
GFDENs: Grapefruit-derived exosome-like nanoparticles
HO-1: Heme oxygenase-1
GDENs: Ginger-derived exosome-like nanoparticles
IECs: Intestinal epithelial cells
LGG: Lactobacillus rhamnosus
TLDENs: Tea leaves-derived exosome-like nanoparticles
BDENs: Broccoli-derived exosome-like nanoparticles
AMPK: Adenosine monophosphate activated protein kinase
MBDENs: Mulberry bark-derived exosome-like nanoparticles
AhR: Aryl hydrocarbon receptor
COPS8: Constitute photomorphogenic homolog subunit 8
CDENs: Carrots-derived exosome-like nanoparticles
TET8: Tetraspanin-8
PEN1: PENETRATION 1
EC1: Extracellular domains 1
TL: Targeting ligand
DSPE-PEG: 1, 2-Distearoyl-sn-glycero-3-phosphoethanolamine-Poly (ethylene glycol)
*E. coli*: *Escherichia coli*
*N. gonorrhoeae*: *Neisseria gonorrhoeae*
*P. Aeruginosa*: *Pseudomonas aeruginosa*
*H. influenzae*: *Haemophilus influenzae*
*B. fragiles*: *Bacteroides fragilis*
*A. muciniphila*: *Akkermansia muciniphila*
*L. paracasei*: *Lactobacillus paracasei*
*V. cholera*: *Vibrio cholerae*
*H. pylori*: *Helicobacter pylori*
*S. typhimurium*: *Salmonella typhimurium*
*A. baumannii*: *Acinetobacter baumannii*
*S. toxin*: *Shiga toxin*
*F. nucleatum*: *Fusobacterium nucleatum*
*B. lactis*: *Bifidobacterium lactis*
*L. rhamnoses*: *Lactobacillus rhamnosus*
*B. ovatus*: *Bacteroides ovatus*
*B. thetaiotaomicron*: *Bacteroides thetaiotaomicron*
*S. enterica*: *Salmonella enterica*
*B. cepacia*: *Burkholderia cepacia*
*S. aureus*: *Staphylococcus aureus*
*MenB*: *B Meningococcal*
*Nm*: *Neisseria meningitidis*
*M. tuberculosis*: *Mycobacterium tuberculosis*
*K. pneumonia*: *Klebsiella pneumoniae*
*S. pneumonia*: *Streptococcus pneumoniae*
