# Editorial Expression of Concern: Emerging role of bacterial outer membrane vesicle in gastrointestinal tract

**DOI:** 10.1186/s13099-024-00646-4

**Published:** 2024-09-23

**Authors:** Cheng‑mei Tian, Mei‑feng Yang, Hao‑ming Xu, Min‑zheng Zhu, Yuan Zhang, Jun Yao, Li‑sheng Wang, Yu‑jie Liang, De‑feng Li

**Affiliations:** 1 Department of Emergency, Shenzhen People’s Hospital (The Second Clinical Medical College, Jinan University; the First Affiliated Hospital, Southern University of Science and Technology), Shenzhen, 518020 Guangdong China; 2 Department of Hematology, Yantian District People’s Hospital, Shenzhen, Guangdong China; 3 Department of Gastroenterology and Hepatology, Guangzhou Digestive Disease Center, Guangzhou First People’s Hospital, School of Medicine, South China University of Technology, Guangzhou, China; 4 Department of Medical Administration, Huizhou Institute of Occupational Diseases Control and Prevention, Huizhou, Guangdong China; 5 Department of Gastroenterology, Shenzhen People’s Hospital (The Second Clinical Medical College, Jinan University; the First Affiliated Hospital, Southern University of Science and Technology), No. 1017, Dongmen North Road, Luohu District, Shenzhen, 518020 People’s Republic of China; 6 Department of Child and Adolescent Psychiatry, Shenzhen Kangning Hospital, No.1080, Cuizu Road, Luohu District, Shenzhen, 518020 People’s Republic of China

.

The Editor-in-Chief would like to alert the readers that concerns have been raised regarding the incorrect references 72–77 cited in this article [1]. The text citing these references is related to nucleic acids present in bacterial extracellular vesicles (EV) and outer membrane vesicles (OMVs), whereas cited references 72–77 are related to studies on mammalian cell-derived exosomes. The authors have not responded to the concerns. Readers are urged to take caution when interpreting the content of this article.

None of the authors responded to any correspondence from the Publisher about this Editorial Expression of Concern.
